# Streptokinase Treatment Reverses Biofilm-Associated Antibiotic Resistance in *Staphylococcus aureus*

**DOI:** 10.3390/microorganisms4030036

**Published:** 2016-09-20

**Authors:** Nis Pedersen Jørgensen, Natalia Zobek, Cindy Dreier, Jakob Haaber, Hanne Ingmer, Ole Halfdan Larsen, Rikke L. Meyer

**Affiliations:** 1 Interdisciplinary Nanoscience Center, Aarhus University, 8000 Aarhus C, Denmark; nisjoerg@rm.dk (N.P.J.); zobeknatalia@gmail.com (N.Z.); cd@inano.au.dk (C.D.); 2 Department of Infectious Diseases, Aarhus University Hospital, 8200 Aarhus N, Denmark; 3 Department of Veterinary Disease Biology, University of Copenhagen, 1870 Frederiksberg C, Denmark; jhaa@sund.ku.dk (J.H.); hi@sund.ku.dk (H.I.); 4 Department of Clinical Biochemistry, Aarhus University Hospital, 8200 Aarhus N, Denmark; ole.halfdan.larsen@clin.au.dk; 5 Department of Bioscience, Aarhus University, 8000 Aarhus C, Denmark

**Keywords:** USA300, biofilm, human plasma, fibrinolysis, MBEC

## Abstract

Biofilms formed by *Staphylococcus aureus* is a serious complication to the use of medical implants. A central part of the pathogenesis relies on *S. aureus’* ability to adhere to host extracellular matrix proteins, which adsorb to medical implants and stimulate biofilm formation. Being coagulase positive, *S. aureus* furthermore induces formation of fibrin fibers from fibrinogen in the blood. Consequently, we hypothesized that fibrin is a key component of the extracellular matrix of *S. aureus* biofilms under in vivo conditions, and that the recalcitrance of biofilm infections can be overcome by combining antibiotic treatment with a fibrinolytic drug. We quantified *S. aureus* USA300 biofilms grown on peg-lids in brain heart infusion (BHI) broth with 0%–50% human plasma. Young (2 h) and mature (24 h) biofilms were then treated with streptokinase to determine if this lead to dispersal. Then, the minimal biofilm eradication concentration (MBEC) of 24 h old biofilms was measured for vancomycin and daptomycin alone or in combination with 10 µg/mL rifampicin in the presence or absence of streptokinase in the antibiotic treatment step. Finally, biofilms were visualized by confocal laser scanning microscopy. Addition of human plasma stimulated biofilm formation in BHI in a dose-dependent manner, and biofilms could be partially dispersed by streptokinase. The biofilms could be eradicated with physiologically relevant concentrations of streptokinase in combination with rifampicin and vancomycin or daptomycin, which are commonly used antibiotics for treatment of *S. aureus* infections. Fibronolytic drugs have been used to treat thromboembolic events for decades, and our findings suggest that their use against biofilm infections has the potential to improve the efficacy of antibiotics in treatment of *S. aureus* biofilm infections.

## 1. Introduction

A key aspect of pathogenesis by *Staphylococcus aureus* is the formation of biofilms [1] by interacting with mammalian extracellular matrix (ECM) molecules on ECM-covered surfaces in the body [2,3]. *S. aureus* possesses an array of cell wall-anchored proteins responsible for these specific receptor-ligand interactions, collectively called “microbial surface components recognizing adhesive matrix molecules” [2]. These include Clumping factor A and B and SdrE which bind to fibrinogen and complement factors [4,5,6], the fibronectin- and fibrinogen-binding proteins FnBPA and FnBPB [7,8,9], the fibronectin-binding proteins Ebh, the collagen-binding Collagen Adhesin (Cna) [10], and many other proteins for which the ligands are yet to be described [2]. In addition to these receptor-ligand interactions, a recent study also demonstrated that cell-surface proteins containing particular domains with internal thioester bonds could react with fibrinogen to form a covalent interaction, and that many pathogens, including *S. aureus*, contain cell surface proteins with similar domains [11].

*S. aureus* biofilm infections are often localized to bones, soft tissues, heart valves [12,13,14], or implants which are quickly covered with ECM molecules following implantation [15,16,17]. After adhesion to ECM molecules, *S. aureus* induces coagulation, which results in the formation of a three dimensional matrix of fibrin around the cells. *S. aureus* secretes two proteins that induce coagulation: coagulase and von Willebrand factor-binding protein. These proteins highjack the coagulation cascade by binding to prothrombin, which results in the formation of staphylothrombin that converts fibrinogen to fibrin. Conversely, *S. aureus* can also induce degradation of fibrin by secreting staphylokinase, which activates plasminogen to plasmin—a serine protease capable of fibrinolysis.

The addition of human plasma facilitates coagulation during growth of *S. aureus* in laboratory media and therefore greatly stimulates biofilm formation in vitro [18,19]. In vivo, the production of coagulase and the fibrin-binding Clumping factor A and FnBPs are reported to be particularly critical for biofilm formation [20]. Along with the reported lack of biofilm formation in staphylokinase-overexpressing strains [21], these findings testify to the key role of fibrin in the *S. aureus* biofilm matrix in vivo.

The formation of a fibrin matrix provides *S. aureus* with an ideal defense mechanism against immune cells [22,23], and *S. aureus* biofilms are also more recalcitrant to antibiotic therapy, both in vitro and in vivo [24,25,26]. The antibiotic dosage required for eradication of a mature *S. aureus* biofilms in vitro are in the range of 256–2048 mg/L for vancomycin, daptomycin, and other relevant anti-staphylococcal drugs, which far exceeds the concentration that can be achieved in vivo [25,27]. This recalcitrance to antibiotic treatment is due to a combination of several factors, including the reduced penetration and lower diffusion coefficient of many antibiotics in the biofilm, which has been demonstrated for *S. aureus* biofilms with, for example, vancomycin, oxacillin, and cefotaxime [28,29].

Due to the low efficacy of antimicrobials against cells within biofilms, a combined approach is gaining increasing interest, where the matrix of biofilms is targeted to disperse the biofilm while simultaneously applying antimicrobials. Enzymatic degradation of matrix polysaccharides or eDNA has been applied with varying success in vitro [30,31,32]. However, the knowledge that host-derived components, particularly fibrin, are more important components of the *S. aureus* biofilm matrix in vivo suggests these might be more efficacious targets.

Only a handful of studies have investigated this idea so far. Activation of plasminogen by fibrinolytic drugs is used to treat blood clots, and the same principle could be applied to dissolve the fibrin of *S. aureus* biofilms. Nemoto et al. were the first to demonstrate that 10,000 U/mL of the fibrinolytic drug Varidase (streptokinase) given concurrently with ofloxacin enhanced the efficacy of antibiotic treatment of in vitro grown *S. aureus* biofilms [33]. Recently, several studies have shown that prevention of fibrin formation leads to decreased biofilm formation and higher antibiotic susceptibility of in vivo grown *S. aureus* biofilms. This was shown by preventing *S. aureus*-mediated coagulation by adding a thrombin inhibitor to jugular vein catheters in mice [34], or by adding human plasminogen to a staphylokinase-overexpressing *S. aureus* strain, which led to fibrin dissolution as a result of staphylokinase-mediated activation of plasminogen [20]. Biofilm formation was also reduced by coating implant surfaces with the human tissue plasminogen activator (tPA), and the biofilms that did form were more susceptible to antibiotics [35]. These are encouraging results for the possibility of preventing biofilm formation to avoid acute infections on new implants. However, the treatment of biofilm infections that occur years after implantation is even more critical. Zapotoczna et al. [19] showed that *S. aureus* biofilms formed in vivo in a catheter inserted in the jugular vein in a rat model could be dispersed ex vivo by treating the biofilm with nattokinase. It is, however, not known if the same effect can be obtained in vivo, and which antibiotic concentration is needed to eradicate the biofilm.

The aim of this study was therefore to determine if *S. aureus* USA 300 biofilms could be eradicated by combining streptokinase with selected antibiotics at physiologically relevant concentrations. We grew *S. aureus* biofilms in a high throughput peg-lid biofilm assay and quantified the amount of biofilm after growth in brain heart infusion (BHI) amended with different concentrations of human plasma. We then determined the effect of streptokinase treatment on biofilm dispersal, and on the minimal biofilm eradication concentration (MBEC) of antibiotics provided as single drugs or in combinations. Finally, confocal laser scanning microscopy visualized the biofilm architecture and viability.

## 2. Materials and Methods

### 2.1. Human Plasma Extraction

Human plasma was generously provided by the Blood Bank, Aarhus University Hospital. Identification of the individual donors was not possible. Donors had signed a generalized consent form and since the blood was not to be used in studies involving screening for undiagnosed conditions, further ethical approval was not necessary. Blood from healthy donors was stabilized in 10 mL tubes (BD Vacutainer, Beckton Dickinson, Albertslund, Denmark) coated with EDTA (1.8 mg EDTA/mL blood). Plasma was separated from whole blood by centrifugation at 6000× *g* for 10 min at 5 °C. Subsequently, plasma from more than 100 different donors was pooled to ensure reproducibility. 

### 2.2. Quantification of Human Plasma’s Effect on Biofilm Formation

*S. aureus* USA300 was stored in 50% glycerol at −80 °C. Prior to each experiment, 1 μL was streaked on Tryptic Soy Agar (TSA) (Sigma-Aldrich, Copenhagen, Denmark) and incubated at 37 °C until colonies formed, and then stored at 5 °C for up to one month. A single colony was used to inoculate 10 mL 3.7% Brain-Heart Infusion broth (BHI) (Sigma-Aldrich, Copenhagen, Denmark) in Erlenmeyer flasks and incubated at 37 °C and subjected to 100 rpm shaking for 16–18 h. The overnight culture was then adjusted to OD_600_ = 5.0 in fresh BHI. 

Biofilms were grown in a modified Calgary Biofilm Device protocol [36]. We used flat-bottom 96-well microtiter plates (NUNC 161093, Thermo Fisher Scientific, Hvidovre, Denmark) and matching peg-lids (NUNC-TSP 445497, Thermo Fisher Scientific). Each peg-lid was pre-conditioned by inserting it into a 96-well plate containing 180 μL/well 3.7% BHI enriched with 50% human plasma for 30 min at 37 °C. Peg-lids were then transferred into a new microtiter plate containing 20 μL of the overnight culture of *S. aureus* USA300 prepared as described above, and 180 μL of BHI enriched with either 0%, 5%, 10%, 25%, or 50% plasma. After 2 h incubation at 37 °C, the peg-lids, now covered with a biofilm, were transferred back to the microtiter plates used for pre-conditioning and incubated for 24 h at 37 °C to allow further growth and maturation of the biofilm. To simplify nomenclature, we will refer to the 2 h biofilm as “immature” and the 2 + 24 h as “mature” in the following sections.

The amount of biofilm was then quantified using the crystal violet assay as follows: Each peg-lid with biofilm was dried at 37 °C for 30–60 min and then transferred to a 96-well plate containing 200 µL/well freshly prepared 0.5% crystal violet solution (Sigma-Aldrich) in demineralized water, and left to stain at room temperature for 10 min. Excess stain was removed from the peg-lid by gently rinsing it three times by submersion in demineralized water. After rinsing, the lid was transferred to a new 96-well plate containing 200 µL/well 96% ethanol. After 10 min, the peg-lid was discarded, and the optical density of the crystal violet-stained ethanol solutions was measured at 585 nm. This experiment was carried out with a minimum of 8 replicates.

### 2.3. Biofilm Dispersal with Streptokinase

The peg-lid with *S. aureus* USA300 biofilm (prepared as described above using BHI with 50% plasma) was transferred to a 96-well plate containing 200 μL/well of freshly prepared (up to 5 min in advance) streptokinase (Sigma-Aldrich) in BHI and incubated for 1 h at 37 °C. The amount of the biofilm was then quantified using the crystal violet assay as described above. Treatment of immature biofilms was performed with 0, 50, 100, and 150 U/mL streptokinase, while mature biofilms were treated with 500 U/mL streptokinase in BHI. This experiment was performed with a minimum of three replicates.

### 2.4. MBEC of Antibiotics Combined with Streptokinase

Mature biofilms were prepared as described above using BHI with 50% plasma or tryptic soy broth (TSB) (Fluka Analytical). Antibiotics were dissolved into BHI, and serially diluted (two-fold in each step) in a 96-well plate, such that concentration ranges were 0–2048 μg/mL for vancomycin (Sigma-Aldrich) and daptomycin (Cubicin, Novartis, Denmark). The concentration of rifampicin (Eramfat, Riemser Pharma GmbH, Greifswald, Germany) was, however, kept constant at 10 μg/mL. Peg-lids with mature biofilms were transferred to the plate containing BHI with antibiotics, or antibiotics with 500 U/mL streptokinase (Sigma-Aldrich), and incubated for 24 h at 37 °C. The combination of antibiotics tested were: vancomycin alone, vancomycin with rifampicin, daptomycin alone, and daptomycin with rifampicin.

After antibiotic exposure, biofilms were rinsed by gently submerging the lid in a 96-well plate containing BHI three times and were then transferred to a “recovery plate” containing 500 U/mL streptokinase solution in BHI, and incubated for 24 h at 37 °C. The absence of viable cells among the bacteria detached from the biofilm during incubation with antibiotics and streptokinase was confirmed by spotting 10 µL to a fresh agar plate and incubating overnight at 37 °C. Bacteria-free pegs were used as blanks, and pegs with biofilms that had not been treated with antibiotics were used as growth controls. After the incubation, peg-lids were discarded and planktonic growth was assessed in the wells by transferring 5 μL from wells without visible growth to TSA and incubating for 24 h 37 °C before assessing the presence of colonies. The experiment was performed with a minimum of eight replicates, and the MBEC value was determined as the antibiotic concentration resulting in the absence of viable cells in the recovery plate in at least five out of eight replicates.

It should be noted that streptokinase is usually not included in the recovery plates in standard MBEC protocols. However, in control experiments we found no growth in the recovery plates, even in the absence of antibiotics. Hence the *S. aureus* cells were so firmly attached in the biofilm that no planktonic growth resulted from transferring the biofilm to fresh BHI. With streptokinase in the recovery media, all controls were positive for planktonic growth (see results section).

### 2.5. Confocal Laser Scanning Microscopy (CLSM)

Microtiter plates for microscopy (Product # 89621, Ibidi, Munich, Germany) were pre-conditioned in 180 μL modified BHI-plasma medium containing 78 µL plasma, 90 µL BHI, and 12 µL Alexa Fluor^®^ 647-conjugated fibrinogen (Thermo Fisher Scientific) in sodium bicarbonate, pH 8.3. The resulting concentration of Alexa Fluor-conjugated fibrinogen was 200 μg/mL, which corresponds to approximately 20% of the total fibrinogen expected to be present in the media. The plates were incubated for 30 min at 37 °C before the addition of 20 μL of overnight culture (adjusted to OD_600_ = 5) was added to each well (final OD_600_ = 0.50, final volume = 200 μL/well) and incubated for 2 h at 37 °C. The medium was then gently removed by aspiration and replaced with 300 μL of the modified BHI-plasma medium. The plate was then incubated for 24 h at 37 °C to generate mature biofilms. The biofilms were then incubated for another 24 h at 37 °C in fresh media (modified BHI-plasma medium), or media amended with 4 μg/mL vancomycin and 10 μg/mL rifampicin, or media amended with 500 U/ml streptokinase in addition to the antibiotics.

CLSM imaging was done with a Zeiss LSM700 confocal laser scanning microscope (Zeiss, Jena, Germany). The medium was removed, and each well was gently rinsed five times in 300 μL sterile phosphate buffered saline (PBS) before adding 150 μL PBS containing 10 μM of the membrane-permeant DNA-binding stain SYTO 41 (Thermo Fisher Scientific) for live-cell imaging, and 2 μM of the membrane-impermeant DNA-binding stain TOTO^®^-1 (Thermo Fisher Scientific) for dead cell imaging. The biofilms were incubated at room temperature for 45–60 min with the staining solution before imaging. SYTO 41 and Alexa Fluor^®^ 647 were captured in one track with excitation by the 405 nm and 635 nm lasers and splitting the emission to the two photomultipliers at 600 nm, and TOTO^®^-1 was then captured by excitation with the 488 nm laser in a second track. 

### 2.6. Statistics

Statistical tests were done using the GraphPad Prism 5.0f (Graphpad Software, La Jolla, CA, USA). The data was plotted to assess normal distribution, and since the data follow a normal distribution, all tests were done using parametric testing (Student’s *t*-test).

First-order and zero order equations were fitted to the data from crystal violet staining of biofilms to determine the dose-response effect of adding human plasma to BHI, and of treating pre-formed biofilms with increasing concentrations of streptokinase.

## 3. Results

### 3.1. Human Plasma Promotes Formation of Biofilms That Can Be Dispersed by Streptokinase Treatment

**Figure 1:**
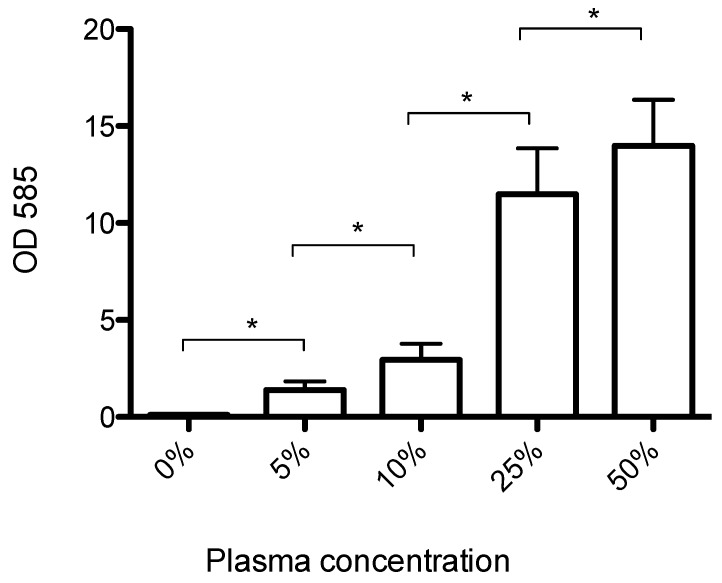
Mean biofilm biomass (±SD, *n* = 8), measured by crystal violet staining. USA300 formed very little biofilm in pure brain heart infusion (BHI), but the addition of human plasma led to increased biofilm formation in a dose-dependent manner. * significantly different (*p* < 0.001).

*S. aureus* USA300 formed very little biofilm in BHI, but the addition of increasing concentrations of plasma led to a dose-dependent increase in biofilm formation (Figure 1). The amount of biofilm formed increased linearly (*R*^2^ = 0.96) at plasma concentrations up to 25% and then leveled off, indicating that the availability of plasma proteins was the limiting factor for biofilm formation at plasma concentrations below 25%. 

**Figure 2:**
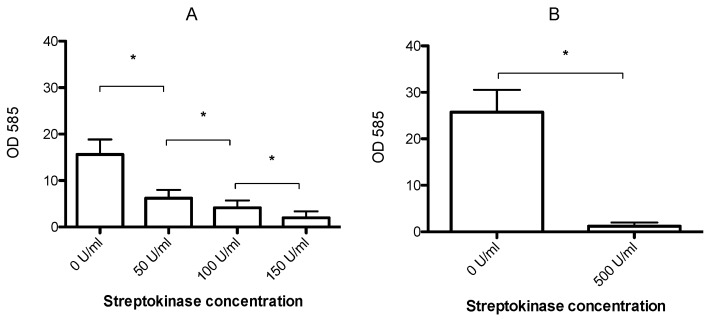
Mean biofilm biomass (± SD, *n* = 8), measured by crystal violet staining after incubation of 2 h old (**A**) and 24 h old (**B**) biofilms with streptokinase for 24 h. Increasing concentrations of streptokinase resulted in a dose dependent decrease in biomass. A single high dose was applied to 24 h old biofilms, resulting in almost complete biofilm dispersal. * significantly different (*p* < 0.001).

Immature (2 h old) biofilms could be partially dispersed by 1 h streptokinase treatment (Figure 2A). The amount of biofilm decreased in a dose-dependent manner after treatment with 50, 100, or 150 U/mL streptokinase (*p* < 0.05). Mature (24 h old) biofilms could also be dispersed, but required higher streptokinase concentrations. Treatment with 500 U/mL lead to a 22-fold reduction of biomass from 25.8 to 1.2 (Figure 2B).

### 3.2. Streptokinase Treatment Lowers the MBEC to Clinically Relevant Levels

The addition of streptokinase during antibiotic treatment dramatically increased the susceptibility of biofilms to antibiotics, resulting in lower MBEC values (Table 1). The most pronounced effect was observed when streptokinase was applied together with a combination of rifampicin and either vancomycin or daptomycin. The combination of streptokinase and two antibiotics lowered the MBEC to a level that can safely be obtained in vivo (Table 1).

**Figure 3:**
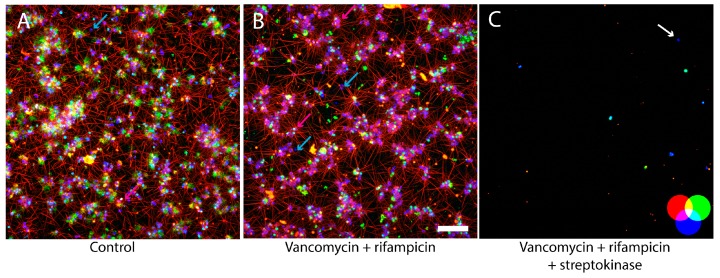
Confocal Laser Scanning Microscopy (CLSM) imaging of USA300 biofilms grown in BHI with plasma and Alexa Fluor conjugated fibrinogen. After 24 h growth, biofilms were incubated for 24 h in media (**A**), or media amended with vancomycin (4 mg/L) and rifampicin (10 mg/L) (**B**), or media amended with vancomycin (4 mg/L) and rifampicin (10 mg/L) and streptokinase (500 U/mL) (**C**). Alexa Fluor conjugated fibrinogen appear **red**, viable cells stained by SYTO 41 appear **blue**, and dead cells stained by TOTO^®^-1 appear **green**, cyan, and **yellow** because some of these cells also bound fibrinogen and SYTO 41. **Blue** arrows indicate cells that did not appear to bind fibrinogen, and **pink** arrows indicate cells that bound fibrinogen and appeared to cause coagulation of fibrinogen to fibrin fibers. Combination of antibiotics with streptokinase resulted in almost complete eradication of the biofilm. A single viable cell that did not bind TOTO^®^-1 is indicated by the arrow. Scale bar = 20 µm.

We hypothesized that fibrin was a major part of the biofilm matrix, and that the effect of streptokinase was due to fibrinolysis. We therefore visualized the biofilm architecture, including the fibrin component. *S. aureus* USA300 biofilms were grown for 24 h in BHI supplemented with human plasma and Alexa Fluor^®^ conjugated fibrinogen before transferring and incubating the biofilms for an additional 24 h in either fresh media, or media supplemented with antibiotics, streptokinase, or both. CLSM imaging showed biofilms consisting of aggregates of cells with fibrin fibers extending more than 20 µm from the cell surface (Figure 3A). The addition of 4 mg/L vancomycin and 10 mg/L rifampicin did not result in visible losses of biomass or viable cells (Figure 3B), but the combination of antibiotics with streptokinase almost completely removed the fibrin and eliminated the biofilm (Figure 3C).

To test if a secondary non-fibrinolytic effect of streptokinase was at play, we grew biofilms in tryptic soy broth in the absence of human plasma and performed MBEC assays with and without streptokinase. These biofilms were deficient of fibrin and other human ECM proteins, and we confirmed that streptokinase had no effect on the MBEC values (Table 2).

## 4. Discussion

This study confirms that host-derived proteins are an integral part of the *S. aureus* USA300 biofilm matrix, and that fibrin provides a target for therapies that can potentiate the efficacy of conventional antibiotics.

The addition of plasma to BHI stimulated biofilm formation immediately, and the large amount of biofilm formed after the 2 h incubation period stresses that most of the biofilm was formed by the aggregation of cells and coagulation of fibrin, rather than by growth. As expected, the biofilms were rich in fibrin (Figure 3A,B), which extended as long fibers from the cell surface. Some cells, but not all, were also coated with fluorescent fibrinogen that did not appear to take a fibrillar form (Figure 3, blue and pink arrows). This cell to cell variation probably illustrates the variability in expression of fibrinogen binding proteins, such as FnBPs, clumping factor, and SdrE. It is also interesting to note that some of the dead cells, which were recognized by their ability to bind the membrane-impermeable stain TOTO^®^-1 (green), also appeared to be a source of fibrin production. Many of these cells appear yellow, as they also bind fibrinogen (red). This observation implies that dead cells may still be a source of coagulase, and that the remains of these cells bind to fibrinogen and fibrin, and thereby become integrated in the biofilm matrix.

Streptokinase only dispersed biofilms grown in BHI with plasma (Table 1 and Table 2), and microscopy confirmed that fibrinolysis was responsible for this effect, as fibrin was completely removed from the biofilm after the addition of streptokinase (Figure 3). Streptokinase works by activating plasminogen to plasmin by proteolysis, and the fibrinolytic effect is therefore dependent on the presence of plasminogen. The treatment of biofilms with antibiotics and streptokinase was performed in BHI without plasma, and the available plasminogen must therefore be bound in the biofilm and transferred from the previous incubation in BHI with 50% plasma. Plasminogen can be bound to *S. aureus* cells by several mechanisms. Pietrocola et al. recently showed that FnBPB is responsible for binding the bulk of plasminogen bound in the *S. aureus* biofilm matrix [37]. Furthermore, the manganese transport protein C is also capable of binding a range of human extracellular matrix and coagulation cascade proteins, including plasminogen [38]. This could explain why a sufficient amount of plasminogen was present to induce fibrinolysis in the biofilm upon activation by streptokinase in BHI. The dose-dependent response to increasing streptokinase concentrations even indicates that streptokinase and not plasminogen was the limiting factor for the biofilm dispersal.

The antibiotic susceptibility of the biofilms appeared to be strongly affected by the composition of the extracellular matrix. MBEC for vancomycin or daptomycin when combined with 10 mg/L rifampicin were only 64 mg/L for biofilms grown in BHI with 50% plasma, but exceeded the 1024 mg/L upper detection limit of the assay when biofilms were grown in TSB. This difference suggests that the MBEC for biofilms in vivo may not be as high as predicted by measurements made in standard laboratory media.

EDTA-stabilized plasma was chosen over heparin-stabilized plasma because previous studies have shown that heparin can induce biofilm formation by *S. aureus* [39]. However, EDTA can also induce adverse effects due to its toxicity. We used EDTA-stabilized plasma with a final EDTA concentration of 0.9 mg/mL in media with 50% plasma, but the concentration of free EDTA was likely much lower, as EDTA would be bound to components in the plasma. We confirmed that EDTA did not inhibit biofilm growth of *S. aureus* USA300, and the concentration of free EDTA must therefore have been below the MBC for this strain. Previous studies found a synergistic effect between antibiotics and EDTA against *S. aureus* biofilms, but these results were obtained at 30 mg/mL EDTA [40,41]. We therefore argue that the theoretical maximum of 0.9 mg/mL EDTA in our study was unlikely to have affected our results.

A natural limitation of our study is the ex vivo conditions, which raises the question of whether one would expect to obtain a similar effect of streptokinase in vivo, because the effect may depend on the biofilm thickness and architecture. Bjarnsholt et al. demonstrated that in vivo biofilms are generally thinner than in vitro grown biofilms, and often contains only a few cell layers [42]. Our in vitro grown biofilms were very thick and easily visible to the naked eye, and would therefore expect that the dose of streptokinase used in our experiment would also be effective on in vivo grown biofilms. We were able to fully disperse the biofilms (Figure 1 and Figure 3) with a dosage of streptokinase that is many times lower than the dosages used to treat patients with thromboembolic events. Hence, even if the dosage used here is insufficient for dispersing in vivo grown biofilms, there is ample room for maneuvering when adapting this approach to in vivo treatment.

An important question is also whether our observations apply generally to *S. aureus*, as the matrix composition can vary from between strains, and the fibrin production is a balance between coagulase activity and staphylokinase activity. In particular, the methicillin-resistant *S. aureus* used in this study is known to rely more on proteins and less on polysaccharides compared to methicillin-sensitive *S. aureus* [43]. However, several recent studies have confirmed the important role of fibrin for methicillin-sensitive *S. aureus* biofilms in vivo [20,34], and that ex vivo treatment with fibrinolytic drugs can disperse biofilms that are formed on implant surfaces in vivo [19].

To the best of our knowledge, our study demonstrates for first time that a drug combination leads to eradication of mature biofilms at antibiotic concentrations that are achievable in patients. Nemoto et al. demonstrated that streptokinase treatment in combination with ofloxacin reduced the number of viable bacteria in *S. aureus* biofilms to less than 10% of tests with antibiotics alone [33]. However, this reduction was only achieved with a streptokinase dosage of 10,000 U/mL and an ofloxacin concentration of 128 mg/L. While streptokinase can be administered at those concentrations, the ofloxacin concentration far exceeds what is clinically possible [44]. Even at this high concentration, biofilm eradication was not observed. 

We therefore hypothesized that the successful non-invasive eradication of *S. aureus* biofilms could be achieved at physiologically relevant antibiotic concentrations if fibrinolytic drugs were combined with the most effective combination of antibiotics. Indeed, we found no effect of streptokinase on the MBEC for daptomycin, and only a 50% decrease of the MBEC for vancomycin when used as single drugs. It was only when combining one of the antibiotics with rifampicin that streptokinase treatment resulted in a dramatic decrease in the MBEC to clinically achievable levels. The maximum recommended dose of daptomycin is 10 mg/kg, which results in a total in vivo concentration of approximately 10–100 mg/L [45]. Current clinical guidelines recommend a trough concentration of vancomycin of between 15–20 mg/L to avoid adverse events [46,47]. By combining a multi-drug treatment, we show here that biofilm eradication could be achieved at 4 mg/L daptomycin or vancomycin. As this concentration is easily achievable in vivo, our study provides an encouraging lead for further investigation of using this drug combination to treat biofilm infections.

Our study demonstrated that good in vitro models are essential for investigating the role of targeting the biofilm matrix to treat biofilm infections. We therefore stress the importance of host-derived proteins for the structure and properties of the biofilm matrix, and propose that human plasma is included as standard when growing *Staphylococcus* biofilms in vitro. If our findings are to be applied to the treatment of biofilms in vivo, the use of fibronolytic drugs in conjunction with antibiotics could improve treatment outcome for *S. aureus* biofilm infections. Prosthetic valve endocarditis caused by *S. aureus* has a one-year mortality rate of 50% and even with surgery performed, re-infection rate is about 6%–15% [48,49]. The need to improve the efficacy of antibiotic treatment is therefore critical, and this study provides an important first step towards this goal.

## 5. Conclusions

In this study we demonstrated that adding human plasma to BHI increased biofilm formation by *S. aureus* USA300. Mature *S. aureus* biofilms could be eradicated with clinically relevant concentrations of daptomycin with rifampicin and vancomycin with rifampicin when treatment was concurrent with clinically relevant dosages of streptokinase.

## Figures and Tables

**Table 1:** Minimal biofilm eradication concentration (MBEC) against biofilms of *S. aureus* USA300 grown for 24 h in BHI with 50% plasma and incubated with antibiotics in the presence or absence of streptokinase. Rifampicin was added as a fixed dosage of 10 mg/L.

Antibiotic	MBEC (−Streptokinase) (mg/L)	MBEC (+Streptokinase) (mg/L)
Daptomycin	1024	1024
Daptomycin + rifampicin	64	<4
Vancomycin	128	64
Vancomycin + rifampicin	64	4

**Table 2:** MBEC against biofilms of *S. aureus* USA 300 grown for 24 h in tryptic soy broth (TSB) and incubated with antibiotics in the presence or absence of streptokinase. Rifampicin was added at a fixed dosage of 10 mg/L.

Antibiotic	MBEC (+Streptokinase) (mg/L)	MBEC (−Streptokinase) (mg/L)
Daptomycin + rifampicin	>1024	>1024
Vancomycin + rifampicin	>1024	>1024
